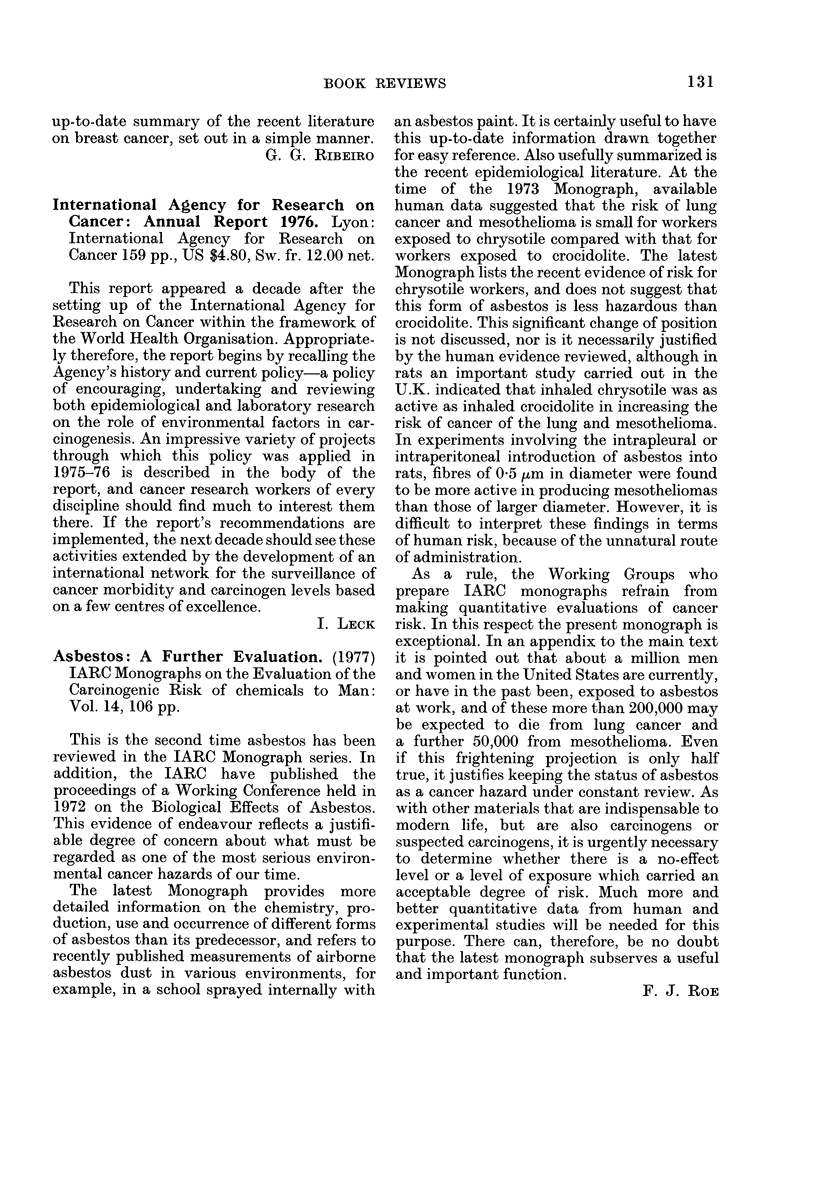# International Agency for Research on Cancer: Annual Report 1976

**Published:** 1978-01

**Authors:** I. Leck


**International Agency for Research on Cancer: Annual Report 1976.** Lyon: International Agency for Research on Cancer 159 pp., US $4.80, Sw. fr. 12.00 net.

This report appeared a decade after the setting up of the International Agency for Research on Cancer within the framework of the World Health Organisation. Appropriately therefore, the report begins by recalling the Agency's history and current policy—a policy of encouraging, undertaking and reviewing both epidemiological and laboratory research on the role of environmental factors in carcinogenesis. An impressive variety of projects through which this policy was applied in 1975–76 is described in the body of the report, and cancer research workers of every discipline should find much to interest them there. If the report's recommendations are implemented, the next decade should see these activities extended by the development of an international network for the surveillance of cancer morbidity and carcinogen levels based on a few centres of excellence.

I. LECK